# Deep Learning-Based Real-Time Multiple-Person Action Recognition System

**DOI:** 10.3390/s20174758

**Published:** 2020-08-23

**Authors:** Jen-Kai Tsai, Chen-Chien Hsu, Wei-Yen Wang, Shao-Kang Huang

**Affiliations:** Department of Electrical Engineering, National Taiwan Normal University, Taipei 106, Taiwan; johnny850219@gmail.com (J.-K.T.); wywang@ntnu.edu.tw (W.-Y.W.); grghwng@hotmail.com (S.-K.H.)

**Keywords:** smart surveillance, action recognition, deep learning, human tracking

## Abstract

Action recognition has gained great attention in automatic video analysis, greatly reducing the cost of human resources for smart surveillance. Most methods, however, focus on the detection of only one action event for a single person in a well-segmented video, rather than the recognition of multiple actions performed by more than one person at the same time for an untrimmed video. In this paper, we propose a deep learning-based multiple-person action recognition system for use in various real-time smart surveillance applications. By capturing a video stream of the scene, the proposed system can detect and track multiple people appearing in the scene and subsequently recognize their actions. Thanks to high resolution of the video frames, we establish a zoom-in function to obtain more satisfactory action recognition results when people in the scene become too far from the camera. To further improve the accuracy, recognition results from inflated 3D ConvNet (I3D) with multiple sliding windows are processed by a nonmaximum suppression (NMS) approach to obtain a more robust decision. Experimental results show that the proposed method can perform multiple-person action recognition in real time suitable for applications such as long-term care environments.

## 1. Introduction

Due to the rise of artificial intelligence in the field of video analysis, human action recognition has gained great popularity in smart surveillance, which focuses on automatic detection of suspicious behavior and activities. As a result, the system can launch an alert in advance to prevent accidents in public places [1] such as airports [2], stations [3], etc. To serve the need of the upcoming aging society, smart surveillance can also provide great advantages to long-term care centers as well, where action recognition can help center staff notice dangers or accidents when taking care of large numbers of patients. Therefore, it is important to develop an action recognition system for real-time smart surveillance.

Recently, data-driven action recognition has become a popular research topic due to the flourish of deep learning [4,5,6,7,8]. Based on the types of input data, existing literature of action recognition can be divided into two categories: skeleton-based and image-based methods. The former includes 3D skeleton [9,10] generated by Microsoft Kinect, and 2D skeleton generated by OpenPose [11] and AlphaPose [12]. The latter includes approaches using single-frame images [13], multiframe video [14,15], and optical flow [16]. Note that the size of skeleton data is much smaller than that of the image data. For example, the size of 3D skeleton data and image data in the NTURGB+D dataset is 5.8 and 136 GB, respectively. It is easy to see that the size of skeleton data is 23 times smaller than that of the image data. Thus, the training process by using skeleton data is faster than that by using image data. However, the image data might contain vital information [17], including age, gender, wearing, expression, background, illumination, etc., in comparison to skeleton data. Moreover, in order to identify each individual appearing in the scene, the face image is also needed in the proposed method. Thus, image-based methods render more information of the scene for wider applications, such as smart identification and data interpretation, which is of practical value and worthy of further investigation.

Convolution neural networks (CNN) is a powerful tool in the field of image-based action recognition. According to the dimension of the convolution unit used in the network, previous approaches can be separated into 2D and 3D convolution approaches. Although 2D convolution provides appealing performance in body gesture recognition, it cannot effectively handle the recognition of continuous action streams. This is due to the lack of temporal information, which imposes the limitation of modeling continuous action with 2D convolution. On the other hand, 3D convolution contains an additional time dimension that can remedy this deficiency. Thus, 3D convolution has been widely used in recent data-driven action recognition architectures [18], such as C3D (convolutional 3D) [19], I3D (inflated 3D ConvNet) [20], and 3D-fused two-stream [21]. Widely seen as foundational research, Ji [14] proposed taking several frames from an input video and simultaneously feeding them into a neural network. Features extracted from the video contain the information in both time and space dimensions via 3D convolution that can be utilized to generate results for action recognition. Although the recognition performance of [14], evaluated based on the TRECVID 2008 [22] and KTH datasets [23], provided good action recognition, the accuracy of 3D convolution relies on a huge amount of network parameters, leading to low efficiency in the recognition process. This inevitably causes difficulty in reaching a real-time action recognition. To solve this problem, a recent approach [21] developed a two-stream architecture, taking both RGB and optical flow images as input data, that improves I3D by adopting the concept of Inception v1 [24] in GoogLeNet. Inception v1 contains 2D convolution using a 1 × 1 filter in the network to reduce the amount of training parameters, and hence improves the efficiency of recognition. As a result, accuracy of the I3D approaches outperforms traditional approaches in the well-known action recognition datasets, such as UCF101 [25] and Kinetics [26].

Although the I3D-based action recognition has a desired performance, the original version of I3D cannot recognize actions of multiple people appearing in a scene at the same time. The I3D-based recognition system also encounters problems when locating the start and end time for each of the actions in the input video for subsequent action recognition [27], because there is likely a mismatch between the start and end time of the actions and the segmented time interval.

In order to solve these problems, we propose a real-time multiple-person action recognition system with the following major contributions. (1) We extend the use of I3D for real-time multiple-person action recognition. (2) The system is capable of tracking multiple people in real time to provide action recognition with improved accuracy. (3) An automatic zoom-in function is established to enhance the quality of input data for recognizing people located far from the camera. (4) The mismatch problem mentioned earlier can be addressed by adopting a sliding window method. (5) To further improve the accuracy, recognition results from I3D with multiple sliding windows are processed by a nonmaximum suppression (NMS) approach to obtain a more robust decision. Experimental results show that the proposed method is able to achieve multiple-person action recognition system in real time.

The rest of the paper is organized are follows: Section 2 introduces the proposed real-time multiple-person action recognition system, Section 3 presents the experimental results, and the conclusion is given in Section 4.

## 2. Real-Time Multiple-Person Action Recognition System

**Figure 1:**
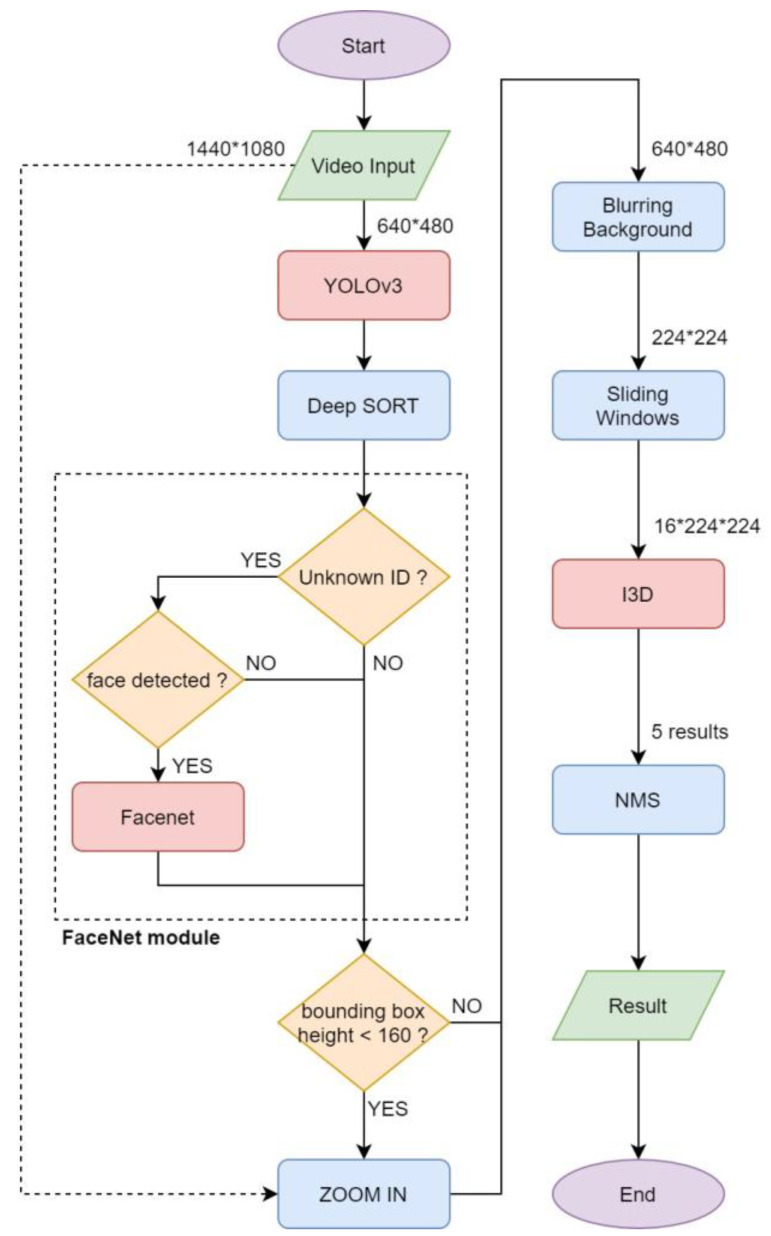
Flow chart of the proposed multiple-person action recognition system (I3D, inflated 3D ConvNet; NMS, nonmaximum suppression).

Figure 1 shows the complete flow chart of the proposed multiple-person action recognition system. First of all, we use YOLOv3 [28] to locate multiple people appearing in the scene. The Deep SORT [29] algorithm is used to track the people and provide each of them with an identity (ID) number. To identify the person’s name for display, FaceNet [30] is then used for face recognition to check whether or not the ID exists. For people far from the camera, we also establish a “zoom in” function to improve the recognition performance. Video frames in sliding windows are preprocessed and resized for being fed into I3D for action recognition. Finally, this system utilizes a one-dimensional NMS to postprocess the outputs from I3D to improve accuracy and robustness of action recognition.

### 2.1. YOLO (You Only Look Once)

Object detection can be separated into two main categories: two-stage and one-stage methods. The former, such as RCNN [31], fast RCNN [32], faster RCNN [33], and mask RCNN [34], detects the locations of different objects in the image at first, and then recognizes the objects. The later, such as YOLO [35] and SSD [36], combines the both tasks through a neural network. YOLOv3 [28] is a neural networks based object detection algorithm implemented in the Darknet framework, which can obtain the class and a corresponding bounding box for every object in images and videos. Compared with previous methods, YOLOv3 has the advantages of fast recognition speed, high accuracy, and capability of detecting multiple objects at the same time [37]. Thus, this paper uses YOLOv3 at the first stage of action recognition for two reasons. The first one is because the system has to locate each of the people appearing in the scene in real time. The other one is because the information of the bounding box corresponding to each person in the scene is critical for preprocessing in the proposed system. In this step, we convert the input video from camera into frames. The frames are then resized from 1440 × 1080 to 640 × 480 for use by YOLOv3 to obtain a detection result represented by coordinates of the bounding boxes. Note that the purpose of the frame-resizing process results from the consideration of speed and accuracy of object detection.

### 2.2. Deep SORT

Simple online and real-time tracking (SORT) is a real-time multiple object tracking method proposed by Bewley [38]. It combines a Kalman filter and Hungarian algorithm, predicting the object location in next frame according to that of the current frame by measuring the speed of detected objects through time. It is a tracking-by-detection method that performs tracking based on the result of object detection per frame. As an updated version of SORT, simple online and real-time tracking with a deep association metric (Deep SORT) proposed by Wojke [29] contains an additional convolutional neural network for extracting additional features, which is pretrained by a large-scale video dataset, the motion analysis and reidentification set (MARS). Hence, Deep SORT is capable of reducing SORT tracking error by about 45% [29]. In the proposed system, the goal of using Deep SORT is to perform multiple-person tracking, where an ID number corresponding to each individual is created into a database based on the detection results of YOLOv3. This means that each person appearing in the scene will be related by an individual bounding box and a ID number.

### 2.3. FaceNet

FaceNet is a face recognition approach revealed by Google [30], which has achieved an excellent recognition performance of 99.4% accuracy in the LFW (labeled faces in the wild) database. It adopts Inception ResNet network architecture and applies a pretraining model based on MS-Celeb-1M dataset. Note that this dataset contains 8 million face images of about 1 million identities. During the pretraining process, it first generates 512 feature vectors via L2 normalization and embedding process. Then, the feature vectors are mapped into a feature space to calculate the Euclidean distance between features. Finally, a triple loss algorithm is applied in the training process. In the processing of FaceNet, the similarity of face-matching depends on calculating the Euclidean distance between the features of the input face image and the stored face images in the database. As soon as the similarity of each matching pair is given, SVM classification is applied to make the final matching decision.

**Figure 2:**
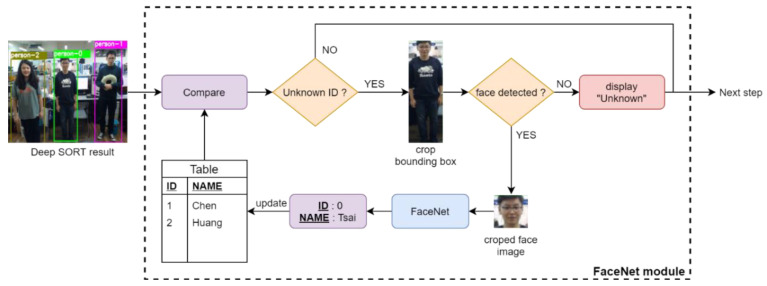
Flow chart of face recognition via FaceNet.

The face recognition process of the proposed method is shown in Figure 2. At the beginning, we prestore matched pairs of individual names and their corresponding features generated by FaceNet in the database. When the bounding box image and corresponding ID number of each individual are obtained from Deep SORT, we check if the ID number exists in the database for reducing the redundant execution of FaceNet. Considering the efficiency for real-time application, we separate the condition as to which individual does not require a face-matching process via FaceNet from others. If the ID number does exist in the database, the name related to the ID number will be directly obtained from the database. Otherwise, we will check whether the face of the individual can be detected by utilizing the Haar-based cascade classifier in OpenCV, based on the bounding box image of the individual. If the face of the individual cannot be detected, FaceNet will not be executed, resulting in an “Unknown” displayed on the top of the bounding box in the video frames. Otherwise, the cropped face image is fed into FaceNet for face-matching by comparing with the stored features in the database. When the feature of the cropped face image is similar to the stored feature in the database, we update the table with the ID number and the corresponding name retrieved from the database for display on the top of the bounding box in the video frames.

### 2.4. Automatic “Zoom-In”

**Figure 3:**
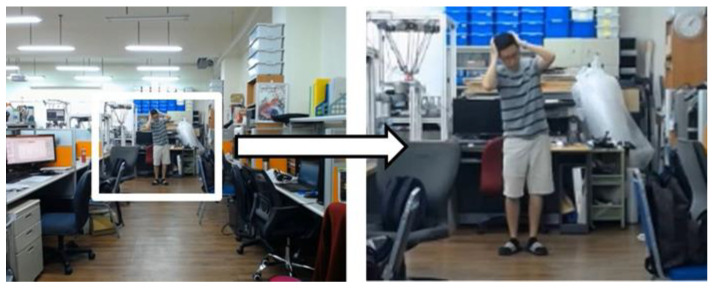
The proposed zoom-in method.

At this stage, every image for further processing has been resized into 640 × 480 pixels, which is much smaller than the original image of 1440 × 1080 pixels. However, if there are individuals located far from the camera, the size of the bounding box in this case will become too small for making accurate action recognition. Fortunately, we are able to utilize the high resolution (1440 × 1080 pixels) of the original video to zoom-in the bounding box for individuals located at a longer distance. Because the position of the camera is fixed, we can roughly estimate the depth of the individual according to the height of the bounding box in the image. If the individual is located far from the camera, the height of the bounding box will be a much smaller value. Here, we set a threshold to determine when to automatically launch the zoom-in function according to the height of the bounding box in the image. Once the zoom-in function is activated, we locate the center of the bounding box in the original 1440 × 1080 image frame and crop a new image of 640 × 480 centered at the bounding box, as shown in Figure 3. Note that the cropped region will not exceed the boundary of the original image.

### 2.5. Blurring Background

**Figure 4:**
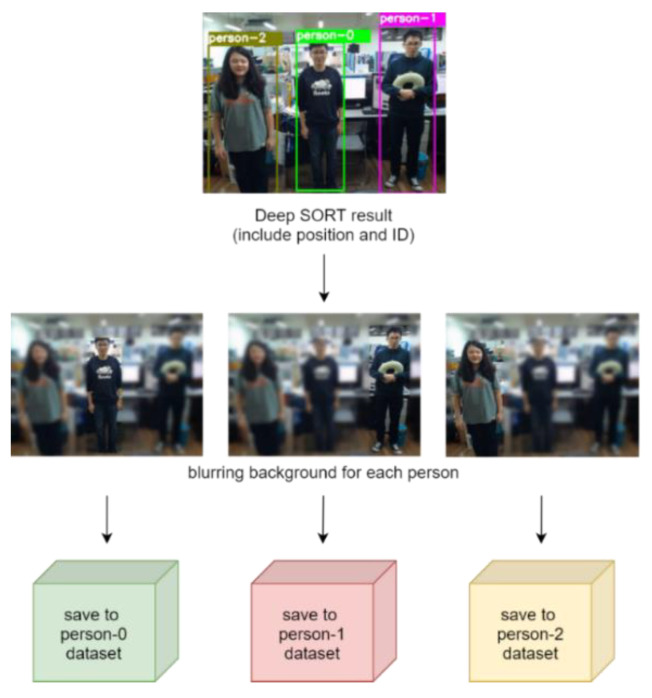
The process of blurring background.

In this process, we aim at utilizing image processing for enhancing the performance of action recognition by I3D. For each individual, we blur the entire image of 640 × 480 except the corresponding bounding box by a Gaussian kernel. Because the image region within the bounding box of each individual contains more important information than the other regions in the image for recognition purpose. In Figure 4, we can see from the top that three people have been detected. Take the individual marked as “person-0” for example. The far left image in the second row in Figure 4 shows that only the region within the bounding box of person-0 remains intact after the blurring process. The same process applies to the other two individuals as well. Then, all of the preprocessed images related to each individual are further reduced into smaller images of 224 × 224 for collection into an individual dataset used by respective sliding windows. The design of this process is to retain important information of the images and reduce interference of the background region for improving recognition accuracy.

### 2.6. Sliding Windows

**Figure 5:**
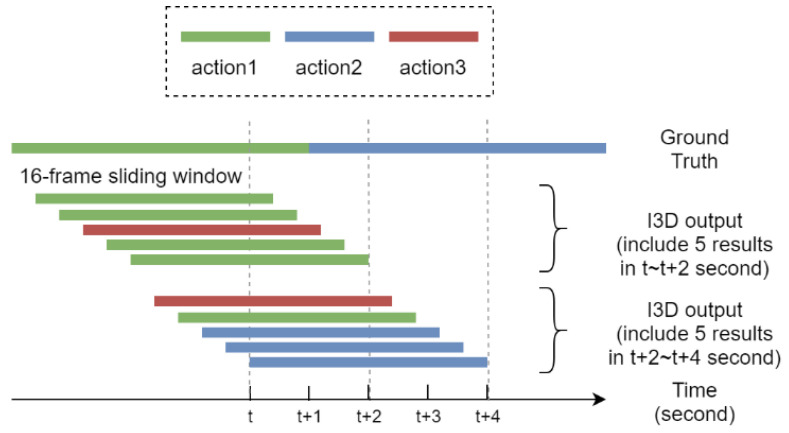
The proposed sliding windows method for action recognition, where from top to bottom shows the ground truth of actions and overlapped sliding windows each consisting of 16 frames.

As mentioned earlier, most work on human action detection assumed presegmented video clips which are then solved by the recognition part of the task. However, the information of the start and end time of an observed action are important in providing satisfactory recognition for continuous action streams. Here, we apply the sliding windows [39] method to divide the input video into a sequence of overlapped short video segments, as shown in Figure 5. In fact, we sample the video with blurred background every five frames to construct a sequence of frames for processing by a sliding window of 16 frames as time elapses. The 16 frames in the sliding window, presumably indicating the start and end time of the action, for each person detected are then fed into I3D to recognize the action performed by the person. Specifically, each video segment *F* consisting of 16 frames in a sliding window can be constructed by:

(1) $$F=\{f_{c-5n}|0\leq n<16\}$$

where *f_c_* is the frame captured at current time. Hence, each video segment representing 80 frames from the camera will be fed into I3D for action recognition in sequence. In this paper, five consecutive sliding windows each consisting of 16 frames and their corresponding recognition class by I3D are grouped as an input set for processing by NMS, as shown in Figure 5.

### 2.7. Inflated 3D ConvNet (I3D)

**Figure 6:**
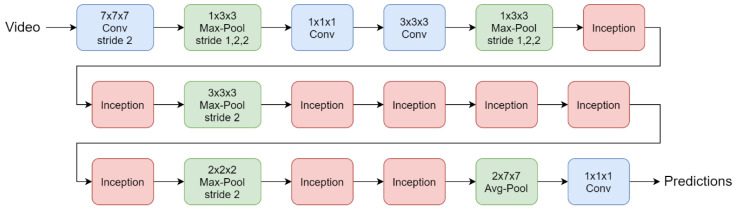
Inflated 3D (I3D) network architecture [20] © 2020 IEEE.

**Figure 7:**
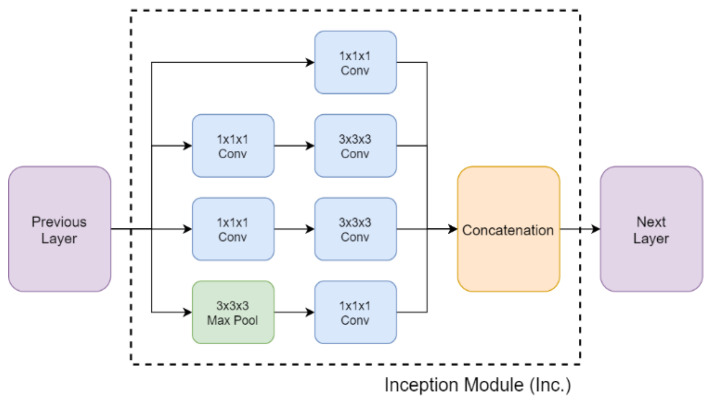
Inception module network architecture [20] © 2020 IEEE.

I3D, a neural network architecture based on 3D convolution proposed by Carreira [20], as shown in Figure 6, is adopted for action recognition in the proposed system, taking only RGB images as the input data. Note that optical flow input in the original approach is discarded in the proposed design considering the recognition speed. Moreover, I3D contains several Inception modules that hold several convolution units with a 1 × 1 × 1 filter, as shown in Figure 7. This design allows the dimension of input data to be adjusted for various sizes by changing the amount of those convolution units. In the proposed method, the input data to I3D are the video segments of a window size of 16 frames from the previous stage. Therefore, each of the video segments is used to produce a recognition class and a corresponding confidence score via I3D based on the input: 16 frames × 224 image height (pixels) × 224 image width (pixels) × 3 channels.

### 2.8. Nonmaximum Suppression (NMS)

Traditional two-dimensional nonmaximum suppression is commonly used for obtaining a final decision of multiple object detection. Basically, NMS generates the best bounding box related to the target object by iteratively eliminating the redundant candidate bounding boxes in the image. To accomplish this task, NMS requires the coordinates of the bounding box related to the individual and the corresponding confidence score. During the iteration, the bounding box having the highest confidence score is selected to calculate the intersection over union (IoU) score with each of the other bounding boxes. If the IoU score is greater than a threshold, the corresponding confidence score of the bounding box will be reset to zero, which means that bounding box will be eliminated. This process repeats until all bounding boxes are handled. As a result, bounding boxes having the highest confidence are selected after the iteration.

**Figure 8:**
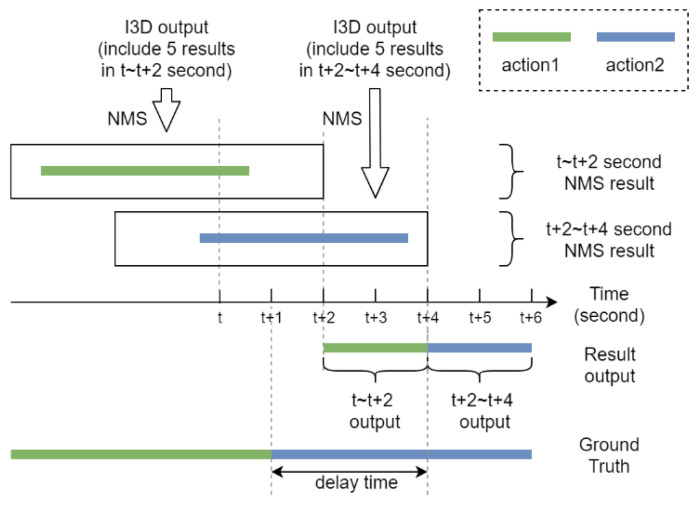
One-dimensional nonmaximum suppression (NMS) is used to derive the final recognition results.

As an attempt to improve the robustness of the recognition results, start and end times of the five consecutive sliding windows and their corresponding recognition class are used to derive the final recognition results via NMS. Figure 8 shows how a one-dimensional nonmaximum suppression is used to derive the final recognition results, where the threshold of the IoU score is set as 0.4. From top to bottom, we can see that each group of five consecutive sliding windows and their corresponding recognition class are utilized to recognize the action class and its best start and end time via NMS among a group of five consecutive sliding windows and their corresponding confidence score. The final recognition result is a winner-take-all decision, resulting in the final estimate of the action class held by the video segment with the highest confidence score. According to Figure 8, we can see that not until the last (fifth) sliding window of frames provides an output does the proposed method make a final estimate of action recognition. Although there is a delay around 2.5 s between the actual recognition results and ground truth, the NMS process can effectively suppress the inconsistency of the action recognition results.

## 3. Experimental Results

### 3.1. Computational Platforms

To evaluate the proposed action recognition system, we train the I3D model on the Taiwan Computing Cloud (TWCC) platform in the National Center for High-performance Computing (NCHC) and verify the proposed method on an Intel (R) Core(TM) i7-8700 @ 3.2GHz and a NVIDIA GeForce GTX 1080Ti graphic card under Windows 10. The computational time of training and testing our I3D model is about 5 h on TWCC. The experiments are conducted under Python 3.5 that utilizes Tensorflow backend with Keras library and NVIDIA CUDA 9.0 library for parallel computation.

### 3.2. Datasets

In this paper, training is conducted on NTU RGB+D dataset [40], which has 60 action classes. Each sample scene is captured by three Microsoft Kinect V2 placed at different positions, and the available data of each scene includes RGB videos, depth map sequences, 3D skeleton data, and infrared (IR) videos. The resolution of the RGB videos is 1920 × 1080 pixels, whereas the corresponding depth maps and IR videos are all 512 × 424 pixels. The skeleton data contain the 3D coordinates of 25 body joints for each frame. In order to apply the proposed action recognition system to a practical scenario, a long-term care environment is chosen for demonstration purpose in this paper. We manually select 12 action classes that are likely to be adopted in this environment, as shown in Table 1. In the training set, most of the classes contain 800 videos, except the class “background” that has 1187 videos. In the testing set, there are 1841 videos.

### 3.3. Experimental Results

In order to investigate the performance of the proposed method for practical use in a long-term care environment, there are five major objectives for evaluation:

- Can the system simultaneously recognize the actions performed by multiple people in real time?
- Performance comparison of action recognition with and without “zoom-in” function;
- Differences of action recognition using black background and blurred background;
- Differences of I3D-based action recognition with and without NMS;
- Accuracy of the overall system.

**Figure 9:**
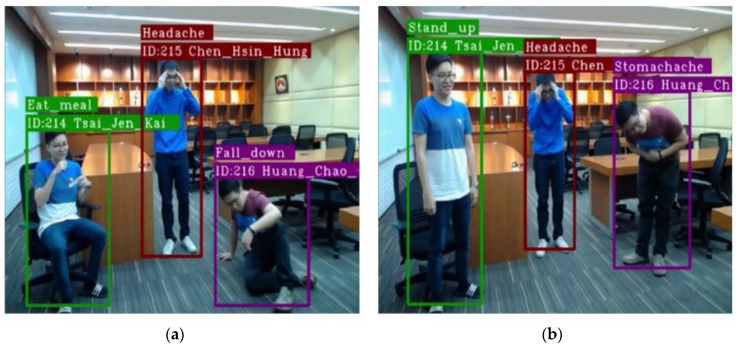
Action recognition results where action classes are (**a**) Eat_meal, Headache, Fall-down (**b**) Stand_up, Headache, Stomachache.

In the first objective, after training with the dataset for 1000 epochs, two screenshots selected from an action recognition result video are shown in Figure 9, where actions performed by three people in a simplified scenario are recognized at the same time. As individuals behave in the scene over time, for example, eating a meal and standing up for ID 214, having a headache for ID 215, and falling down and having a stomachache for ID 216, the system can recognize the actions corresponding to what the individuals are performing. In addition, the proposed system can also display the ID and name obtained by Deep SORT and FaceNet, respectively. There is no hard limit of the number of people appearing in the scene for action recognition, as long as the individuals can be detected by YOLO in the image. However, if the bounding box image of an individual is too small, the system might encounter a recognition error. Table 2 shows the execution speed of the proposed system for various numbers of people, including facial recognition and action recognition.

**Figure 10:**
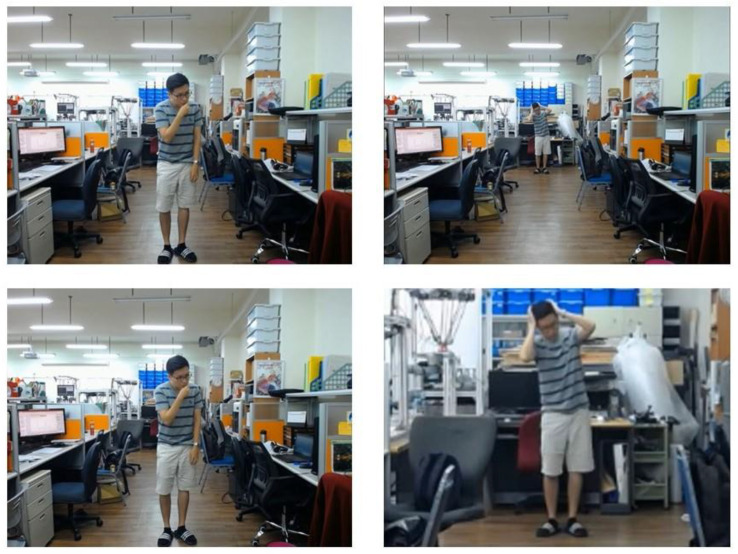
Images of a video where a person moves backward from near (**left**) to far (**right**) to evaluate the recognition performance with and without zoom-in method.

**Figure 11:**
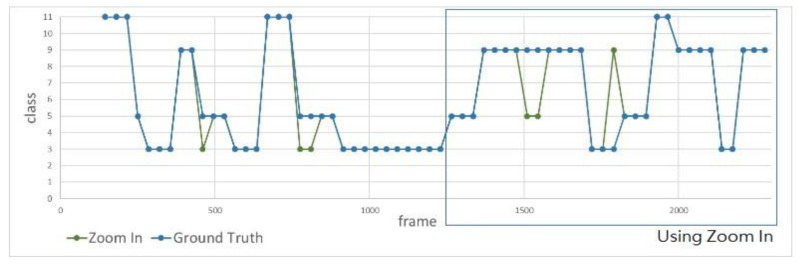
Recognition result using the zoom-in function (green line), where the blur rectangle indicates the individual is located far from the camera.

**Figure 12:**
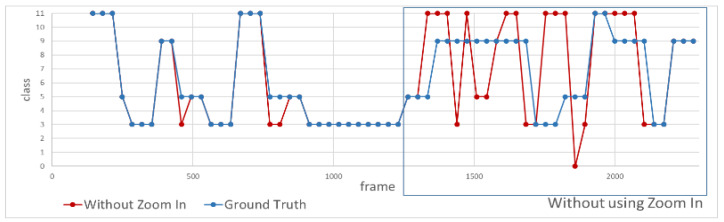
Recognition result without the zoom-in function (red line), where the blur rectangle indicates the individual is located far from the camera.

Second, we design an experiment to measure the action recognition results with and without using the zoom-in function. Figure 10 shows the individual facing the camera slowly moves backward while doing actions. It can be seen that as time elapses, the individual will move farther and farther away from the camera, resulting in poor recognition performance because the size of the bounding box is too small to make accurate action recognition. On the contrary, the bounding box becomes sufficiently large with the room-in function as shown in the bottom-right image of Figure 10. Figure 11 and Figure 12 show the recognition results with and without using the zoom-in function, where the horizontal axis indicates the frame number of the video clip and the vertical axis reveals the action class recognized with the zoom-in function (green) and without the zoom-in function (red), respectively, in comparison to the manually labeled ground truth (blue). Table 3 shows the recognition accuracy for the same individual standing at different distances. We can see that the average accuracy of the recognition results with and without the zoom-in method is 90.79% and 69.74%, respectively, as shown in the far left column in Table 3. As clearly shown in these figures, we can see that there is no difference of the recognition results with or without the zoom-in function for the individual located near the camera. However, when the individual moves far from the camera, the zoom-in approach greatly enhances the recognition accuracy from 32.14% to 89.29%.

**Figure 13:**
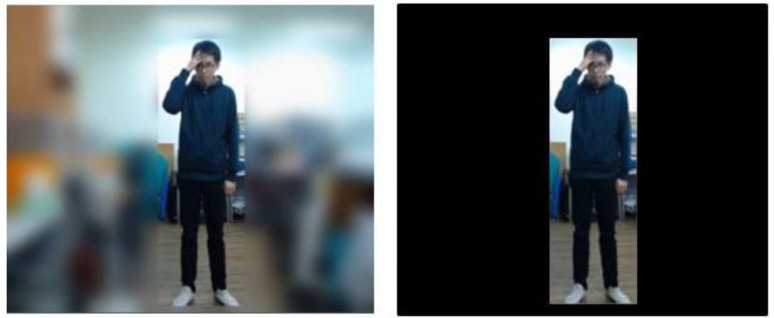
Images with blurred background (**left**) and black background (**right**).

**Figure 14:**
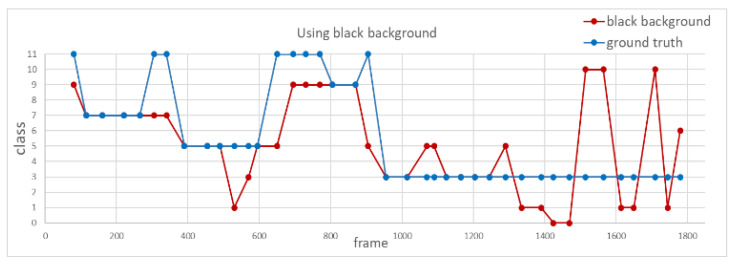
Recognition result using blurred background.

**Figure 15:**
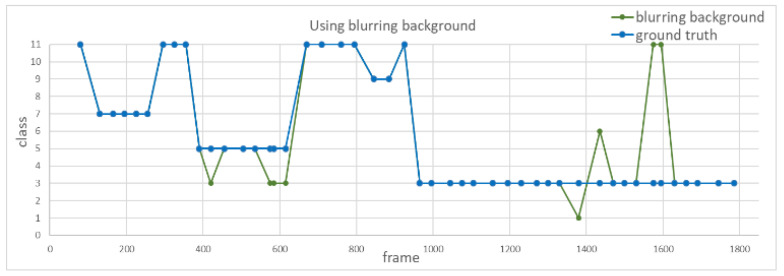
Recognition result using black background.

Third, to investigate the difference for image frames with blurred background and black background for action recognition, we use the same video input to analyze the accuracy of action recognition with different background modifications, as shown in Figure 13. Figure 14 and Figure 15 show the recognition results using blurred and black backgrounds, respectively. It is clear that the recognition result is better by using blurred background (green line) than black background (red line), in comparison to the manually labeled ground truth (blue), where the recognition accuracy of using images with blurred background is 82.9%, whereas the one with black background is 46.9%. We can see that retaining appropriate background information contributes to a better recognition performance.

**Figure 16:**
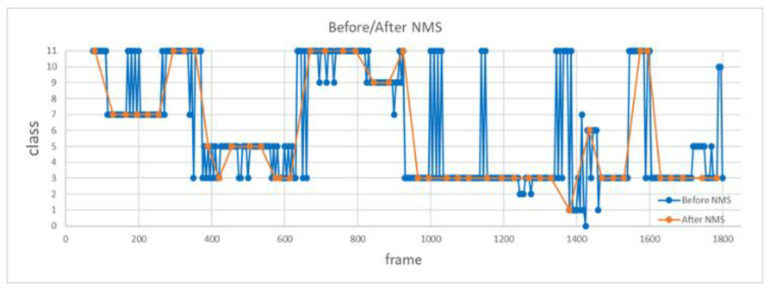
Comparison of recognition results with and without using NMS.

Fourth, to verify the improvement of introducing NMS, we analyze the accuracy of the action recognition system before and after using NMS. In this experiment, we fed a video of 1800 frames consisting of 12 actions into the proposed system for action recognition, as shown in Figure 16, where the orange line and the blue line represent the recognition results with and without using NMS. It is clear that inconsistency of recognition results is greatly suppressed by the NMS method. Note that the average recognition accuracy increases from 76.3% to 82.9% with the use of NMS.

**Figure 17:**
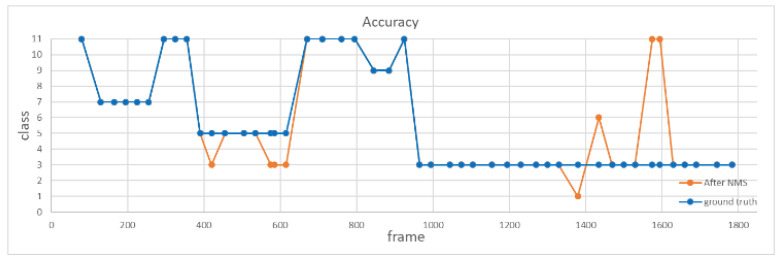
Performance of action recognition of the overall system.

Finally, to show the performance of the proposed approach, Figure 17 shows the recognition accuracy of the overall system, where the orange and blue lines indicate the action recognition results obtained by the proposed method and the manually labeled ground truth, respectively. We can see that except for few occasions, the recognition results are consistent with the ground truth at an accuracy of approximately 82.9%. Due to the fact that this work specifically chose 12 action classes to perform multiple-person action recognition for long-term care centers, there is no universal benchmark for serving the need of a meaningful comparison.

While conducting the experiments, we found that there are some difficult scenarios for action recognition by the proposed system. If two different actions involve similar movement, it may easily incur recognition error. For example, an individual leaning forward could be classified as either ‘Stomachache’ or ‘Cough’ action. Also, if occlusion occurs when capturing the video, the action recognition system is likely to make an incorrect decision. Interested readers can refer to the following link: https://youtu.be/t6HpYCjlTLA to watch a demo video showing real-time multiple-person action recognition using the proposed approach in this paper.

## 4. Conclusions

This paper presented a real-time multiple-person action recognition system suitable for smart surveillance applications to identify individuals and recognize their actions in the scene. For people far from the camera, a “zoom in” function automatically activates to make use of the high resolution video frame for better recognition performance. In addition, we leverage the I3D architecture for action recognition in real time by using sliding window design and NMS. For demonstration purpose, the proposed approach is applied to long-term care environments where the system is capable of detecting (abnormal) actions that might be of concerns for accident prevention. Note that there is a delay of around 2.5 s between the recognition results and the occurrence of observed action by the proposed method. However, this delay does not make significant impacts on the smart surveillance application for long-term care centers, because human response time after the alarm is generally longer than the 2 s delay time. Ideally, a smaller delay time is more appealing. It is our future work to decrease the delay time via optimizing the filter size of the sliding window to achieve the objective of less than 1 s for the delay time. Thanks to the architecture of the proposed method, the proposed method can be used in the future for various application scenarios to provide smart surveillance, for example, detection of unusual behavior in a factory environment.

## Figures and Tables

**Table 1:** Classes of action recognition.

Number	Action	Number	Action
1	Drink	7	Chest ache
2	Eat meal	8	Backache
3	Stand up	9	Stomachache
4	Cough	10	Walking
5	Fall down	11	Writing
6	Headache	12	Background

**Table 2:** Execution speed of the proposed system for various numbers of people.

Number of People	1	2	3	4	8	10
**Average speed (fps)**	12.29	11.70	11.46	11.30	11.02	10.93

**Table 3:** Recognition accuracy with and without the zoom-in method of the scenario in Figure 10.

	Average Accuracy	Short-Distance Accuracy	Long-Distance Accuracy
**With zoom-in**	90.79%	91.49%	89.29%
**Without zoom-in**	69.74%		32.14%
